# Cost-effectiveness of wastewater-based environmental surveillance for SARS-CoV-2 in Blantyre, Malawi and Kathmandu, Nepal: A model-based study

**DOI:** 10.1371/journal.pgph.0004439

**Published:** 2025-04-24

**Authors:** Mercy Mvundura, Lucky G. Ngwira, Kabita Bade Shrestha, Reshma Tuladhar, Jillian Gauld, Cliff Kerr, Kayla Barnes, Catherine Anscombe, Bhawana Sharma, Nicholas Feasey

**Affiliations:** 1 Medical Devices and Health Technologies, PATH, Seattle, Washington, United States of America; 2 Malawi-Liverpool Wellcome Programme, Kamuzu University of Health Sciences, Blantyre, Malawi; 3 Health Economics and Policy Unit, Kamuzu University of Health Sciences, Blantyre, Malawi; 4 Environment and Public Health Organisation, Kathmandu, Nepal; 5 Central Department of Microbiology, Tribhuvan University, Kathmandu, Nepal; 6 Institute for Disease Modeling, Bill & Melinda Gates Foundation, Seattle, Washington, United States of America; 7 Broad Institute, Boston, Massachusetts, United States of America; 8 Liverpool School of Tropical Medicine, Liverpool, United Kingdom; 9 Harvard School of Public Health, Boston, Massachusetts, United States of America; 10 School of Medicine, University of St. Andrews, St. Andrews, United Kingdom; Mahidol University, THAILAND

## Abstract

Wastewater-based environmental surveillance (ES) has been demonstrated to provide an early warning signal to predict variant-driven waves of pathogens such as severe acute respiratory syndrome coronavirus 2 (SARS-CoV-2). Our study evaluated the potential cost-effectiveness of ES for SARS-CoV-2 compared with clinical testing alone. We used the Covasim agent-based model of COVID-19 to simulate disease transmission for hypothetical populations in Blantyre, Malawi, and Kathmandu, Nepal. We simulated the introduction of a new immune-escaping variant over 6 months and estimated health outcomes (cases, deaths, and disability-adjusted life years [DALYs]) and economic impact when using ES to trigger a moderate proactive behavioral intervention (e.g., increased use of masks, social distancing) by policymakers versus no ES and hence a delayed reactive intervention. Costs considered included for ES, clinical testing, treatment, and productivity loss for the entire population due to implementation of the behavioral intervention. We calculated the incremental cost-effectiveness ratios and compared these with local willingness-to-pay thresholds: $61 for Malawi and $249 for Nepal. We performed sensitivity analyses to evaluate the impact of key assumptions on the results. Costs are reported in 2022 US dollars. We estimate that if ES were implemented, approximately 600 DALYs would be averted in Blantyre and approximately 300 DALYs averted in Kathmandu, over the six-month period. Considering health system costs, ES was cost-effective in Blantyre and cost-saving in Kathmandu. Cost-effectiveness of ES was highest in settings with low clinical surveillance, high disease severity, and high intervention effectiveness. However, from the societal perspective, ES may not be cost-effective depending on the magnitude of population-wide productivity losses associated with the proactive behavioral intervention and the cost-effectiveness threshold. SARS-CoV-2 ES has the potential to be a cost-saving or cost-effective tool from the health system perspective when linked to an effective public health response. From the societal perspective, however, the length of the behavioral intervention and its consequences for productivity losses of the entire population may make ES not cost-effective. Implementing ES for multiple pathogens may improve its cost-effectiveness.

## Introduction

The COVID-19 pandemic, caused by severe acute respiratory syndrome coronavirus 2 (SARS-CoV-2), disrupted health systems and economies globally, starting in 2020. It is estimated there were over 775 million COVID-19 cases globally [1] and at least 7 million deaths [2] as of May 2024, which also led to large negative economic impacts. During the height of the pandemic, many high-income countries had robust systems of site-based and latterly at-home testing, either with molecular diagnostic tests (e.g., polymerase chain reaction (PCR)) or rapid antigen tests, respectively [3–6]. However, in many low- and middle-income countries (LMICs), a relatively low proportion of infections were diagnosed with such tests [7] and cases were likely under-reported to health authorities. Furthermore, as of early 2024, even countries that had implemented detailed test-based surveillance of COVID-19 have since discontinued these programs [8].

Thus, alternative surveillance platforms were needed to identify rapidly rising COVID-19 cases and provide information to trigger interventions to control the spread of dangerous new variants. Early in the pandemic, interventions largely consisted of widespread rollout of clinical testing and social lockdowns. However, the social lockdowns had huge negative impacts on economies [9] and could not be sustained as a method to mitigate the transmission of the virus [10,11]. In addition, mass screening in the asymptomatic population (such as through the test-trace-isolate programs) was deemed to be an unsustainable surveillance strategy given resource limitations [9–12].

The capability to maintain community-based surveillance in between waves of SARS-CoV-2 was desired but lacking in most LMICs [13,14]. It is well established that there was a lag between community circulation of SARS-CoV-2 and a subsequent increase in hospitalizations [14,15], and in the absence of community-based clinical surveillance, this window of opportunity to intervene before hospitalizations started to climb was typically missed in LMICs. Since SARS-CoV-2 had a doubling time as short as several days, a rapid response was essential for interventions to have had maximum impact, an effect observed empirically in South Korea [16,17] and New Zealand [18]. Especially in LMICs, there was therefore a need for innovative strategies that were not only low cost but also effective at signaling a rise in transmission or an introduction of a new variant. Such strategies, if linked to proactive implementation of public health interventions to flatten the epidemic curve, had the potential to limit the impact of SARS-CoV-2 on morbidity, mortality, and productivity losses.

Environmental surveillance (ES) has been used for epidemiological surveillance of pathogens in the past and has been of particular value in the polio eradication endgame [19,20]. ES was used to monitor transmission of SARS-CoV-2 starting early in the pandemic, and there is evidence that the virus was detected from wastewater prior to population case peaks detected through clinical surveillance systems [14,19,21–26]. Therefore, ES can provide an early warning signal for rising community transmission [15,27]. Further, ES can be used to sustain community-based surveillance in between waves of SARS-CoV-2. While not replacing diagnostic testing, use of wastewater-based ES for SARS-CoV-2 is expected to be cheaper [28] and more logistically feasible than mass population screening and could complement clinical testing and other interventions, particularly when monitoring for variants of concern [19].

With the availability of COVID-19 vaccines and immunity due to prior infections, SARS-CoV-2 waves have declined in severity and in number of infections. However, ES may still be required as a monitoring tool now that the COVID-19 pandemic has moved to the post-pandemic phase [15]. This approach could also be used for epidemiological surveillance for multiple vaccine-preventable pathogens of public health concern, both endemic and epidemic. There is a dearth of evidence on the potential economic value of ES in LMICs [19,20,29], which may differ in terms of catchment population, costs of ES program activities, and capacity for community-based surveillance. This paper reports on the potential cost-effectiveness of ES as an add-on tool for SARS-CoV-2 monitoring for variants of concern in two LMIC settings that were conducting wastewater and clinical surveillance for SARS-CoV-2: Blantyre, Malawi, and Kathmandu, Nepal. This study aims to provide evidence to policymakers as we prepare for future variant-driven waves of pandemic-causing pathogens similar to SARS-CoV-2 that can be detected through wastewater-based ES.

## Methods

### Environmental surveillance process

ES for SARS-CoV-2 involves four main steps: (1) samples are collected from wastewater (e.g., sewers, wastewater treatment plants) or rivers; (2) these samples are then concentrated, typically in the laboratory; (3) ribonucleic acid extraction is then performed; (4) pathogen detection is done using quantitative real-time PCR, and (5) where it could provide additional information, whole-genome sequencing is performed. The context for conducting ES for SARS-CoV-2 in Blantyre [24] has been described previously and the same process was applied to Kathmandu [28]. Briefly, for the pilots conducted in Blantyre and Kathmandu, wastewater was collected in Blantyre (which has a population of approximately 1 million people) from an initial 7 collection sites, which was scaled up to 80 sites, over the period from May 1, 2020, until December 31, 2021. Weekly samples were analyzed at a laboratory facility at the Malawi-Liverpool Wellcome Programme within Blantyre. For the Kathmandu Valley (with a population of 2.7 million people), weekly ES sample collection commenced in July 2021 and continued until December 2021 across 23 sampling points. Sample processing and analysis were conducted in the Central Department of Microbiology laboratory at Tribhuvan University, Kirtipur, Kathmandu.

### Disease transmission model

SARS-CoV-2 transmission was modeled using the Covasim model (version 3.1.6) [30], which is a stochastic, agent-based SEIRS model. Covasim is fully open source; all model code and documentation are available via http://covasim.org. The simulation begins when a hypothetical variant is introduced in the simulated susceptible population (S). The simulation is run for a hypothetical 6 months, corresponding to the average time between major variant introductions during the first 2 years of the COVID-19 pandemic. (Although the simulations always begin with the introduction of a new variant for simplicity, the epidemic usually ends well within the 6-month window, but the ES system and intervention is held constant throughout; this is comparable to running a much longer simulation in which the ES system is in place and new variants are introduced stochastically with an average interval of 6 months.)

The transmissibility of the introduced variant is assumed to be equal to wild type. This default assumption balances the competing factors that (a) new SARS-CoV-2 variants have much higher baseline transmissibility than wild type, but (b) almost the entire population has some immunity (due to vaccination, infection, or both). Since it is not known either how transmissible or how immune escaping a hypothetical new variant would be, this assumption balances these two factors. Infected agents/individuals are initially in the exposed state (E). After the incubation period, they enter the infectious state (I). These agents can experience either symptomatic or asymptomatic infections. Symptomatic infections begin in the presymptomatic phase and can move through stages of mild, severe, and critical infection, with outcomes at any stage leading to recovery (R), which confers temporary immunity, after which agents return to the susceptible state (S). Critical infections can also lead to death. The model parameters that determine these rates have been published previously, including age-dependent susceptibility, severity, and mortality rates [30]. In addition to using the respective population sizes for the Blantyre and Kathmandu Valley catchment areas, United Nations Population Division data on age structure and household size were also used to initialize the respective Covasim models for Blantyre and Kathmandu.

Since simulations are stochastic, 1,000 repeats of each parameter set are run for a total of 52,000 simulations for each country (half with ES, half without). Simulations were run using Python 3.10 on a cluster of Azure virtual machines. Each simulation takes approximately 3–5 seconds to run, for a total of roughly 140 hours of CPU time. All code is available at https://github.com/amath-idm/covid_es.

### Surveillance system and scenario parameters

The potential outcomes after the introduction of a variant are determined by the implementation of a surveillance system. Surveillance systems are included in the model for both clinical and ES methods. These surveillance systems determine detection of and responses to the introduced variant as well as subsequent outcomes in the population. The parameters for these systems (and other model parameters) are listed in Table 1. The model assumes that clinical surveillance (including PCR and rapid antigen tests) is present in all simulations but limited, and we explore outcomes by whether or not ES was implemented and how the availability of clinical surveillance impacts the outcomes.

**Table 1:** Scenario parameters used for both countries.

Parameter	Description	Default	Sensitivity
ES sampling frequency (days)	How frequently a sample is taken from each ES site (days between samples)	3	1, 14
ES system lag (days)	Minimum number of days between ES sample collection and implementation of an intervention	4	2, 10, 21
Number of ES samples	How many individual samples are taken at each ES sampling site each time sampling is performed	9	1, 3, 100
ES intervention trigger	How many positive ES samples are required to trigger the intervention	1	2, 5
Threshold of ES detection	Minimum disease prevalence threshold for detection through the ES system	0.35%	0.035%, 1%, 3%
Sensitivity of ES detection	How likely a positive detection is from ES when the prevalence is above the threshold	84%	4%, 100%
Clinical testing probability	Proportion of SARS-CoV-2 infections that are diagnosed through clinical testing	0.3%	0%, 0.1%, 1%, 3%, 10%
Clinical intervention trigger	How many positive clinical samples are required to trigger the intervention	30	5, 10, 100
Efficacy of the intervention	Reduction in transmission probability after the intervention is implemented	20%	10%, 30%

Abbreviations: ES, environmental surveillance; SARS-CoV-2, severe acute respiratory syndrome coronavirus 2.

Most of the parameters to characterize the ES system were obtained from the pilot sites and also from [14].

Due to lack of country-specific data to inform inputs on the health impact from ES, the parameters that differed in the model between Malawi and Kathmandu were (a) the population size, and (b) the population age structure. While the age structure has a small impact on the mortality rate (Nepal’s population is older than Malawi’s), the main effect is due to the difference in population size: Kathmandu’s larger population provides more opportunities for disease detection, both through clinical and environmental surveillance.

Clinical surveillance is parameterized by the probability of clinical diagnosis, which represents the proportion of simulated infections that are detected through clinical testing. Testing occurs among people with symptomatic infections, and people who have not tested have an equal probability of testing each day (which in practice means a roughly 3-to-4-day average delay in testing for scenarios in which testing rates are low). In addition, the threshold response parameter determines the number of clinical cases detected on a single day required to trigger an intervention.

Our simulated ES system tracks individuals who are infectious, regardless of whether the infection is symptomatic or is detected through clinical surveillance. Due to uncertainty regarding duration of SARS-CoV-2 survival in wastewater systems, we assume that viral load in the environment is directly reflective of the number of individuals who are infectious on that day. Because we do not incorporate geospatial features into the model, we assume the ES system catchment is the entire population being modeled. The key parameters that determine the ES system are the sampling rate (how frequently samples are being taken), the threshold of detection (the minimum number of individuals shedding to detect a positive sample), the number of ES samples taken during each visit to a wastewater collection site, the sensitivity of the ES system (the probability of a positive sample to be detected), and the number of detections required to trigger an intervention by policymakers. Note that during the pilots described in the previous section, we had 7 pilot sites increasing to 80 sites in Blantyre and 23 sites in Kathmandu; this was oversampling during the pilot phase and these numbers were subsequently reduced to a handful of the most informative sites considering the sustainability of the surveillance beyond the research pilot phase. Therefore, in the model we evaluate 9 sites as the base case and then conduct sensitivity analysis on the implications of having as few as 1 to as many as 100 sites.

Depending on the clinical and ES detections and trigger thresholds, an intervention may or may not occur during the simulation. We model a nonspecific intervention (e.g., increased use of masks, social distancing) that represents a policy response to reduce SARS-CoV-2 spread in the community, and we assume this intervention has a modest effect on transmission (10% to 30% decrease in transmission). We explicitly do not model the impact of lockdowns (which could potentially have a larger impact on transmission), as these would unlikely be implemented immediately following the detection of a new variant. While the intervention being modeled is nonspecific, it corresponds most closely to a response to a respiratory pathogen (i.e., a behavior change that could be implemented as a policy by a government). Interventions for non-respiratory pathogens would likely have costs and time courses different to those modeled here. We assume that once implemented, the intervention stays in place for the duration of the simulation. While it would be more realistic to assume that the intervention is lifted if detected cases (either via clinical surveillance or ES) fall below a certain level, the implementation of a responsive controller would require several additional unknown parameters, and is beyond the scope of the current study.

The model produces estimates of mild and severe COVID-19 cases, hospitalizations, and deaths. Each of these is used to calculate costs from the health care system perspective. We compare these health outcomes without ES (status quo, with only clinical testing) with outcomes when ES was implemented to complement clinical testing. We also assume a delay between when the ES samples are collected and the implementation of an intervention to account for the time required for sample transportation and analysis.

### Cost inputs

We performed the analysis from both the health system and limited societal perspectives. The health system perspective accounts only for costs that could be borne by the health system such as costs for implementing the ES system and direct health system costs associated with seeking health care for SARS-CoV-2 illness. The societal perspective also includes the productivity losses borne by households, in addition to the health system costs. Specifically, the health system costs included direct costs of implementing the ES program. These costs were obtained from a prior study conducted during the pilots in both countries and that study estimated the cost per month of conducting ES, accounting for the costs of consumables, labor, equipment, and transport for each ES activity, including field sampling, concentration, pathogen extraction, and detection. These costs have been reported elsewhere [28] and the values used in this analysis are shown in Table 2.

**Table 2:** Costing and economic parameters (2021 US dollars).

	Value	Data source
**Malawi**		
Cost per clinical COVID-19 test	$1	Assumption from local data
Treatment cost for mild COVID-19	$2	Assumption from local data
Treatment cost for severe COVID-19	$43	Assumption from local data
Base cost for ES per month	$5,476	Ngwira et al. [28]
Additional cost to process each ES sample	$58	Ngwira et al. [28]
**Nepal**		
Cost per clinical COVID-19 test	$13	Assumption from local data
Treatment cost for mild COVID-19	$10	Assumption from local data
Treatment cost for severe COVID-19	$211	Assumption from local data
Base cost for ES per month	$8,470	Ngwira et al. [28]
Additional cost to process each ES sample	$86	Ngwira et al. [28]
**Disease and disutility assumptions**		
Duration of mild COVID-19 infection	7 days	Wolfel et al [31]
Duration of severe COVID-19 infection	15 to 28 days	Verity et al [32]
Disutility value of acute symptomatic COVID-19	0.2	[34–37]
Disutility value of long COVID-19 (first 6 months)	0.05	Culter [38]
Disutility value of long COVID-19 (permanent)	0.001	[34–37]
Proportion of infections that are symptomatic	70%	Davies et al. [42]
Proportion of infections that lead to long COVID-19	10%	Assumption
Productivity loss during mild acute COVID-19	10%	Assumption
Productivity loss during severe acute COVID-19	50%	Assumption
Productivity loss due to policy intervention	10%	Assumption
Productivity loss due to death	100%	Assumption

Abbreviations: ES, environmental surveillance; GDP, gross domestic product.

As mentioned, this analysis also included direct health system costs for disease-related costs for health care, including costs for clinical testing, costs for treating mild cases at home, and costs of inpatient care for severe cases. Costs for care were obtained from secondary data and expert option, as shown in Table 2. We used average treatment costs and multiplied this by the length of hospital stay to estimate the hospitalization costs of severe cases. We assume a mild case lasts 7 days [31] and a severe case from 15 to 28 days [32]. For the societal perspective, we account for productivity loss for the individual during the period of illness and also for the productivity losses for the entire population as a result of the behavioral interventions implemented by policymakers as a result of the ES early warning signal (with ES) or the rising clinical cases (with no ES). Productivity per day was valued at the annual per capita gross domestic product (GDP) of each country [33] divided by 365 days in the year. Costs are presented in 2022 US dollars.

### Cost-effectiveness analysis

Parameters for the costing and economic model are provided in Table 2. A wide range of COVID-19 disutility weights have been published in the literature [34–38]. We used a disutility value of 0.2 as an approximation of the median value used in other studies. Disability-adjusted life years (DALYs) were calculated as the sum of years of life lost (YLL) and years of life with disability (YLD). YLL was calculated as the difference between age at death for each agent in the model who died of COVID-19 and life expectancy at birth assuming full health (estimated at 84 years). YLD was calculated as the sum of the products of disutilities and durations for each agent in the model. In order to capture lifetime effects beyond the duration of the simulation time (e.g., long COVID), incidence rather than prevalence DALYs are used (i.e., all YLD are accrued at the point of infection, and all YLL are accrued at the point of death).

The model’s primary outcome was the cost per DALY averted. We calculated the incremental cost-effectiveness ratio (ICER) as the ratio of costs and DALYs averted with ES relative to no ES. The ICER was compared with the local willingness to pay thresholds of $61 per DALY averted [39] for Malawi and $249 per DALY averted for Nepal [40].

ES does not directly impact DALYs but instead changes the number of days for which the intervention is applied (i.e., if ES provides an early warning signal, the intervention will be applied earlier and potentially for longer). Due to the large stochastic variability in simulations, to determine the relationship between ES and DALYs averted, we first performed a linear regression between the number of days the intervention was applied and the number of DALYs averted. Then, rather than use the actual DALYs from a given simulation (which is subject to significant statistical noise since it is influenced by small differences in the number of deaths), statistical DALYs were used based on this regression relationship. This approach to variance minimization produces comparable results to running a much larger number of simulations.

The health systems cost was calculated as the sum of the costs for clinical testing, ES, and treatment. Additional societal costs included lost productivity due to COVID-19 and lost productivity due to the intervention. No discounting was done as the analysis had a 6-month time horizon.

Although it is difficult to quantify the productivity loss due to a nonspecific intervention, we conservatively estimate that the productivity loss is of roughly the same magnitude as the intervention efficacy (e.g., an intervention with an efficacy of 10% would lead to a roughly 10% productivity loss). While a full analysis of the economics of COVID-related interventions is beyond the scope of this paper, the conceptual argument for a linear relationship as a conservative estimate is that (a) an intervention with zero impact on society would have zero impact on transmission, and (b) an intervention that prevented all transmission (e.g., prevented all human contact) would have severe economic repercussions. In practice, productivity loss is almost certainly less than the reduction in transmission. However, while changes in mobility were well quantified during the COVID pandemic, neither the resultant transmission reduction nor economic impact have been determined with precision, making the ratio of these two quantities even more uncertain.

If there are no societal costs associated with the intervention, then the optimal policy would be to have the intervention in place all the time as a preventative measure, regardless of whether or not any COVID-19 cases are detected. However, such a policy would be unfeasible in the real world. Thus, we include a productivity loss associated with the intervention to capture this tradeoff between minimizing transmission (which requires more stringent interventions [41] and minimizing societal disruption (which requires less stringent interventions). Given uncertainty in this variable, we explored the implications of varying the assumed magnitude of the productivity loss relative to the intervention efficacy.

### Ethical review for the study

The cost-effectiveness study presented in this article was a modeling study that relied on secondary data and so did not require ethics approval. We leveraged data from a costing study conducted by the same researchers and that costing study had received ethical approval. The costing study was determined to not be human studies research by PATH Research Determination Committee and did not need United States ethics committee oversight. The Malawi College of Medicine (now KUHES) Research Ethics Committee exempted the costing study from further ethical review, as no human samples were involved, but used the existing waiver for the ongoing environmental surveillance (P.07/20/3089). The Nepal Health Research Council (NHRC) exempted the study from further ethical review, as no human samples were involved as well.

## Results

### Time series

**Fig 1:**
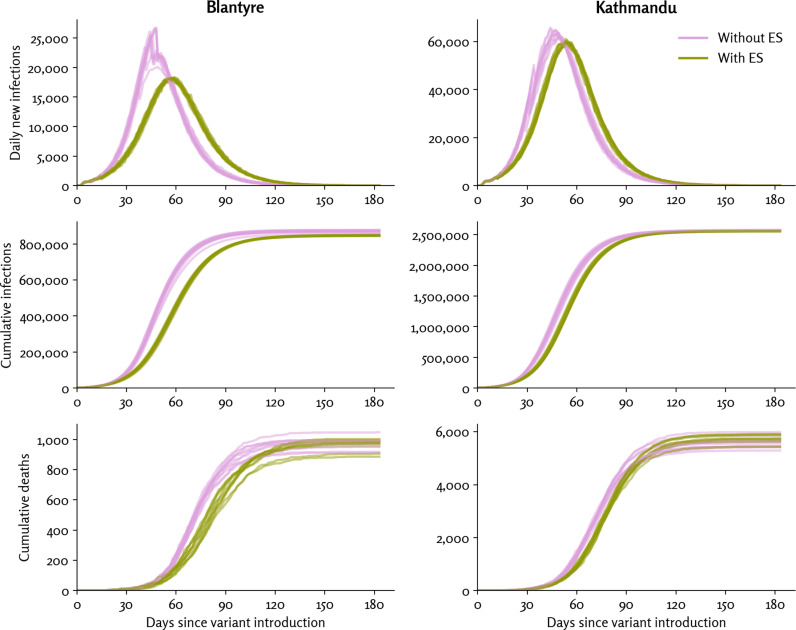
Examples of simulation time series of the default scenario with ES (green) and without ES (purple), for 10 different random seeds. Note that Blantyre and Kathmandu have different y-axis scales.

Example simulation time series for new infections, cumulative infections, and cumulative deaths, both with and without ES for the default set of parameters, are shown in Fig 1. Compared with simulations without ES, simulations with ES have a lower and slightly delayed peak in the number of new infections. However, the difference in cumulative infections and cumulative deaths is smaller. In other words, the intervention primarily has the effect of delaying rather than preventing infections. Time series with and without ES in the absence of clinical surveillance is shown in S1 Fig.

### Days of intervention and DALYs

**Fig 2:**
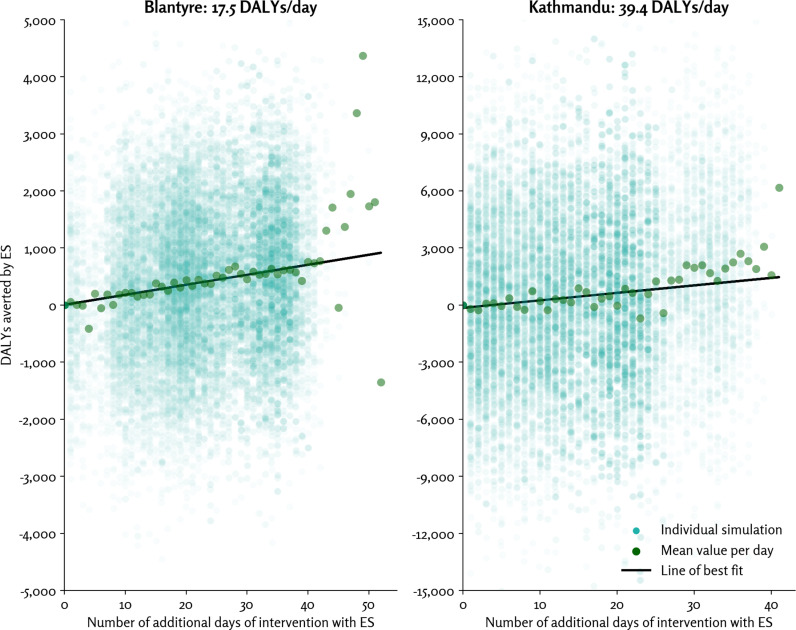
Relationship between the number of additional days in the simulation that the intervention is active when ES is used (x-axis) and the number of DALYs averted (y-axis). For example, a simulation for Malawi would lie on the line of best fit if the intervention started 10 days earlier due to ES, and this simulation had 175 fewer DALYs compared with the same simulation without ES. Note that Blantyre and Kathmandu have different axis scales. Abbreviations: DALY, disability-adjusted life year; ES, environmental surveillance.

The relationship between the number of days the intervention is applied and the number of DALYs averted is shown in Fig 2. The variance between simulations (roughly ±4,000 DALYs for Blantyre and ± 10,000 DALYs for Kathmandu) is much larger than the impact of the intervention; however, a consistent linear trend is still observed. For Blantyre, an average of 17.5 DALYs were averted for each additional day the intervention is applied; for Kathmandu, this value was 39.4 DALYs per day. For Blantyre, the intervention triggered on day 42 ± 10 without ES, and day 21 ± 11 with ES. For Kathmandu, the intervention triggered on day 30 ± 9 without ES, and day 18 ± 8 with ES.

For Blantyre, the ES intervention started a median of 20 days earlier (interquartile range: 10, 31 days); for Kathmandu, it was 10 days (interquartile range: 0, 19 days). As noted previously, the linear relationship between DALYs averted and the number of days the intervention is applied is used to compute the DALYs shown in the results below. The difference is because the clinical and the ES detection triggers were the same in both cases; Kathmandu, with a larger population size, tends to reach the trigger points sooner than Blantyre (for both clinical and ES).

### DALYs

**Fig 3:**
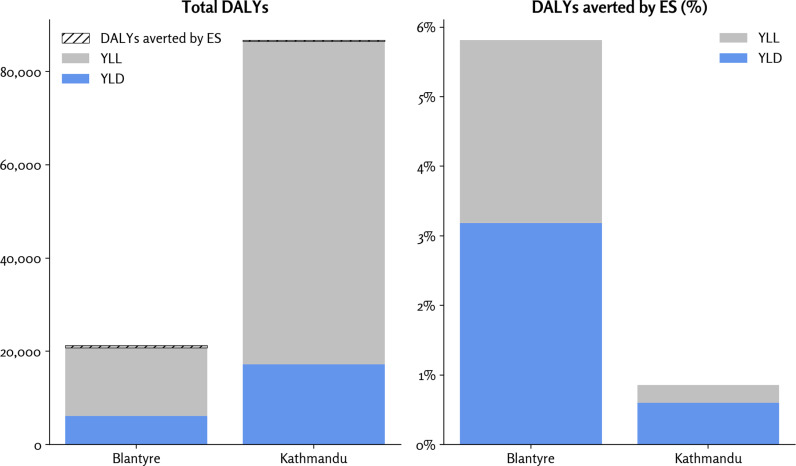
Total DALYs incurred due to COVID (left), and percentage of DALYs averted by ES (right). Abbreviations: DALY, disability-adjusted life year; ES, environmental surveillance; YLD, years of life with disability; YLL, years of life lost.

Our analysis found that DALYs were driven primarily by deaths occurring as measured by years of life lost, although cases not leading to deaths as measured by years of life with disability were a significant proportion as well (Fig 3). ES could avert approximately 6% of total DALYs compared to without ES in Blantyre, and slightly under 1% total DALYs averted in Kathmandu. This large difference between the two countries is primarily due to the 10-day difference in the average number of additional days the intervention is applied with ES (20 for Blantyre and 10 for Kathmandu). Ten days corresponds to roughly two serial intervals for SARS-CoV-2, which, for reproductive number (*R*_*0*_*)* of 2.5, would correspond to roughly a factor of 6 difference in the number of infections.

### Costs and cost-effectiveness

**Fig 4:**
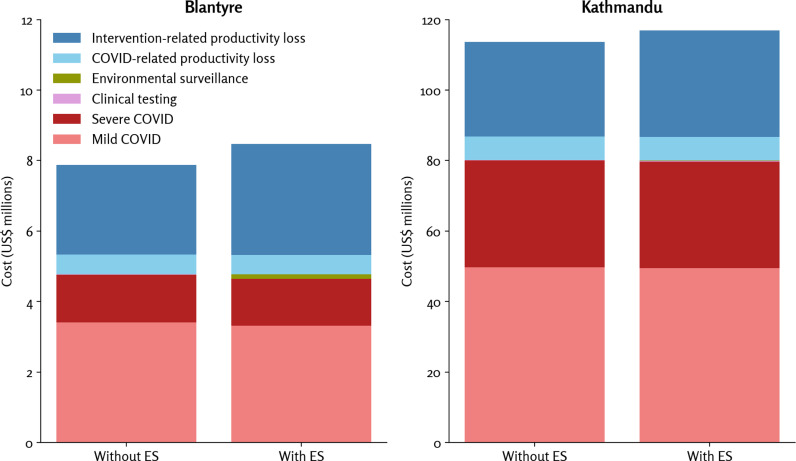
Estimated costs due to COVID, the surveillance programs, and productivity loss. Note the factor of 10 difference in scales between Blantyre and Kathmandu, which is largely due to Kathmandu’s roughly three-times-larger population size and roughly three-times-higher per capita GDP than Blantyre. Note: These data are also listed in Table 3. Abbreviation: ES, environmental surveillance.

Costs are shown in Fig 4. Direct health system costs were driven primarily by the costs of treating mild and severe COVID-19 cases; ES program costs were a small proportion of the total costs (not shown in the table but were $128,622 for Blantyre and $194,860 for Kathmandu), and the cost of clinical testing was relatively small given low prevalence of testing (not shown in the table but these costs were $4,656 for Blantyre and $176,908 for Kathmandu).

**Table 3:** DALYs, DALYs averted, costs, and ICERs.

Outcome	Blantyre				Kathmandu			
	Without ES	With ES	Difference	ICER	Without ES	With ES	Difference	ICER
Total DALYs	21,303	20,709	594		86,777	86,497	279	
Total health system costs	$4,770,034	$4,777,626	$7,591	$12 (cost-effective at all thresholds)	$80,188,952	$80,081,814	–$107,138	$–383 (cost-saving at all thresholds)
Total costs for the societal perspective	$7,875,263	$8,472,803	$597,539	$1,005 (not cost-effective)	$113,711,614	$116,981,874	$3,270,259	11,717 (not cost-effective)

Note: The local cost-effectiveness threshold for Malawi was $61 per DALY averted, and $249 for Nepal.

Abbreviations: DALY, disability-adjusted life year; ES, environmental surveillance; ICER, incremental cost-effectiveness ratio.

For societal costs, the estimated productivity loss from the intervention implemented by policymakers to flatten the epidemic curve was larger than the direct health system costs, since these productivity losses applied to the entire population for the duration of the intervention rather than only to those individuals experiencing symptoms.

Excluding societal costs, the costs saved by preventing COVID-19 infections due to the early warning signal from ES roughly equaled the costs of the ES program, so ES was roughly cost-neutral. Including societal costs, ES had a small net cost, although this was driven entirely by the productivity loss of the intervention rather than the ES program itself.

Overall DALYs, costs, and ICERs are shown in Table 3. Considering only health system costs, ES was very cost-effective in Malawi ($12 per DALY) and cost-saving in Nepal. Considering societal costs, ES was not cost-effective in either Malawi or Nepal.

### Sensitivity analysis

**Fig 5:**
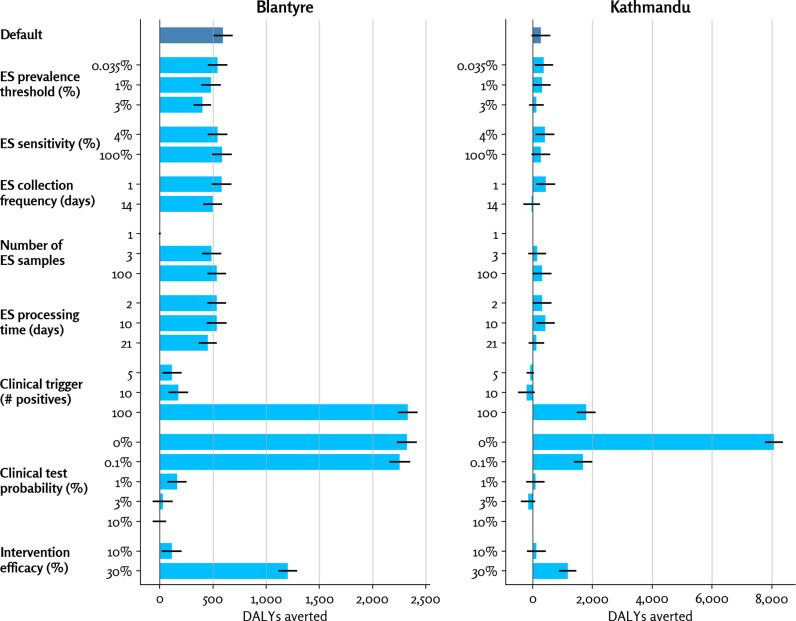
Sensitivity analysis of model parameters. Bars show standard errors of the mean. Values for the base case parameters values are listed in Table 1. Note that Blantyre and Kathmandu have different x-axis scales. Abbreviations: DALY, disability-adjusted life year; ES, environmental surveillance.

To explore the impact of different parameters in the model, we performed a sensitivity analysis (Fig 5). In each case, all parameters are held constant at the default values except for the parameter being varied (see Table 1). Only DALYs are reported here since the variance in DALYs between sensitivity scenarios was much greater than the variance in costs.

The model was most sensitive to the clinical test probability and the clinical trigger threshold. With a low probability of clinical testing or a high trigger threshold, in many cases the intervention is never triggered clinically. ES triggers the intervention 1–3 weeks earlier than the clinical trigger in cases of low clinical testing or a high clinical trigger. The DALYs averted by ES increase significantly if the clinical test probability is below 1% (i.e., fewer than 1% of all SARS-CoV-2 infections are ever diagnosed through clinical testing). Conversely, having a low clinical trigger, meaning a quick and early reaction to few clinical cases, reduces the marginal benefit of ES.

Intervention efficacy also had a relatively large and nonlinear impact on estimated DALYs averted. Increasing the intervention efficacy from 10% to 30% increased the number of DALYs averted from 112 to 1,203 in Malawi and 126–1,176 in Nepal, increases by factors of 11 and 9, respectively. This supralinear increase is due to the fact that larger intervention efficacies can disrupt entire chains of transmission; conversely, small intervention impacts are more likely to delay rather than prevent transmission.

Notably, the results were relatively insensitive to the parameters of the ES system, implying that in the context of low or absent clinical testing, the implementation details of the ES program are less important than whether or not it is present, particularly in a system with widespread outbreak potential, large catchment, and population-wide intervention, represented in our simulated scenarios.

In our default scenario, we assumed that productivity loss was equal to the intervention efficacy (i.e., the intervention reduced transmission by 10% and reduced GDP by 10%). However, our sensitivity analysis showed that a more optimistic assumption about productivity loss could result in ES being cost-effective. In both Malawi and Nepal, our analysis found that for ES to be cost-effective under the local willingness-to-pay threshold would require the productivity loss to be less than 10% of the intervention efficacy (e.g., a transmission reduction of 10% due to the proactive behavioral intervention and a productivity loss of less than 1%).

## Discussion

This study evaluated the potential cost-effectiveness of ES to inform implementation of public health response to the emergence of a new variant of SARS-CoV-2 in Blantyre, Malawi, and Kathmandu, Nepal, to provide evidence on the potential role for ES in LMICs where availability of community testing for pathogens such as SARS-CoV-2 is low. These settings could benefit from lower-cost interventions such as wastewater-based ES, which can operate independently of, but complement clinical testing and provide information on asymptomatic and presymptomatic cases, and those individuals not seeking care at health institutions. Our evaluation found that ES could be cost-saving or cost-effective from the health system perspective. However, it may not be cost-effective from the societal perspective given that the productivity losses associated with the behavioral policy response to flatten the curve affect the entire population. Our finding is an inversion of the usual outcome (i.e., interventions that are sometimes not cost-effective from the health system perspective can be cost-effective from the societal perspective); our finding is due to the fact that the assumed intervention implemented to flatten the epidemic cure (mask wearing, social distancing, and other behavioral interventions implemented to flatten the curve) is applied to the entire society, rather than only people affected by the disease.

However, ES could be cost-effective under more optimistic assumptions where there are smaller productivity losses associated with ES-triggered interventions (for example, masking). Therefore, our results were sensitive to baseline clinical testing, intervention efficacy, magnitude of productivity loss with ES interventions, and ES system costs. From the health system perspective, ES could be a useful tool in LMICs, as the early warning signal may provide policy makers with information to implement preparedness measures which can help flatten the curve, but which will also impact on the health system in other ways for example due to staff redeployment and cancellation of elective medical procedures.

ES is useful if and only if there are pathogens present to detect. In this analysis, we assumed that new variants appeared every 6 months on average (based on four major variants during the first two years of the COVID-19 epidemic). More frequent variant emergence (or other causes of outbreaks) would lead to improved cost-effectiveness for ES, while less frequent outbreaks would lead to lower cost-effectiveness, since the costs of the ES program are ongoing even in the absence of an outbreak. The future of ES is to deploy multi-pathogen platforms to maximise the generation of actionable information for public health services from a single sample, and hence increase the likelihood of ES being cost-effective. Specifically, ES can be used to detect other pathogens of public health importance, including polio and typhoid and can be used as an ongoing monitoring tool. There is considerable potential to deploy multiplex assays at small additional cost, which could significantly improve cost-effectiveness of ES [20,21,24,26]. Here, we focused on one of the most straightforward use cases: deployment of ES for SARS-CoV-2 control interventions upon detection of a variant of concern.

We modeled an Omicron-type variant that was less severe than earlier SARS-CoV-2 variants due to availability of interventions such as vaccines and increasing vaccination rates, and previous population exposure. We found that when simulating an ES system in addition to a clinical surveillance system, the epidemic curve flattened but did not entirely prevent an epidemic due to (a) the infectiousness of the modeled variant, and (b) the assumption of only a relatively modest behavioral change intervention (equivalent to a mask policy) implemented after detection. Despite early warnings, highly infectious pathogens are often established before interventions are implemented, which is seen through our simulation. Modeling more severe variants could have improved the value proposition of ES, where ES can be an even more important addition to disease surveillance and can provide an early warning signal which can reduce transmission and reduce health care and societal cost. In addition, ES may also be a cost-effective intervention for diseases that have a higher burden among younger members of the population (unlike COVID-19), as DALYs averted would be larger when disease is averted among younger populations.

Our analysis also found that the value for money from ES differed between the two countries we modeled. This finding was partly because of significantly higher health care costs in Nepal than in Malawi but a lesser magnitude of difference in health impact (fewer disease cases and deaths; hence DALYs averted). As a result, the cost-effectiveness of ES was lower in Nepal than in Malawi. The implication of this finding is that cost-effectiveness of ES is improved when health care and program costs are lower and disease burden is higher.

This study found that ES provides better value for money in countries where clinical testing rates are low, as ES can provide an early warning signal before people start presenting with COVID to health care facilities. Also, the more effective the proactive behavioral intervention implemented by policymakers to flatten the curve is, the more ES provides value for money. As the COVID-19 pandemic progressed and disease severity declined, policymakers were less likely to implement more stringent but potentially more effective interventions such as lockdowns. If, however, the population does not adhere to the behavioral policy response made by policymakers, the ES signal is not useful as there is no behavior change and no flattening of the curve.

Our findings from the health system perspective are aligned with previous studies. A previous study evaluated the feasibility of ES for SARS-CoV-2 for Ethiopia [29] and another study evaluated the potential cost-effectiveness of ES for Japan [43], but the comparator for the latter study was based on alternative interventions such as screening and was not focused on the value of the early warning signal provided by ES. Another study evaluated the cost-effectiveness of ES for typhoid and found it to be potentially cost-effective [20]. None of the existing studies have evaluated the cost-effectiveness of ES from the societal perspective to include the costs associated with the proactive policy response to the early warning signal, and thus our study provides evidence not existing in the literature.

Our study has several limitations. First, there is considerable uncertainty in some of the parameters used in the model as many economic and health consequences are not yet known [44]. Second, we were unable to obtain country-specific disease burden data and had to rely on assumptions based on global data. Our ES system model assumes a large central catchment for sewage and interventions on the population level, which would not be generalizable to more spatially heterogeneous pathogens or outbreak scenarios. We additionally modeled the impact of ES in urban areas and did not evaluate the potential value in less populous geographies. Thus, our results may not apply to less populous areas of the country. Lastly, there are generalizability issues as we modeled similar interventions in both countries due to lack of country-specific information on what interventions local policymakers would implement to flatten the curve due to an early warning signal from ES. We therefore modeled the impact of behavioral responses with differing impacts to shed light on how this might impact the model findings. We focused on a single variant and intervention scenario; the cost-effectiveness of ES from the health system and societal perspectives could be improved if more severe variants were modeled or other interventions such as vaccines did not exist or were less effective in reducing disease burden.

In conclusion, we found that ES for SARS-CoV-2 has the potential to be a cost-saving or cost-effective tool from the health system perspective when linked to a proactive and effective public health response. From the health system perspective, ES can provide an early warning signal before the population starts presenting with disease cases in health institutions. From the societal perspective, however, the length of the behavioral intervention and its consequences on productivity losses for the entire population may make it not cost-effective. Our simulations do not take into account the potential for using ES to detect multiple pathogens and thus inform public health response to multiple pathogens, which could further improve the cost-effectiveness of ES. This may be an area that could benefit from further research.

## Supporting information

S1 Fig Simulation time series with and without environmental surveillance in the absence of clinical surveillance. (DOCX)

S1 Checklist Inclusivity in global research checklist. (DOCX)

## References

[1] World Health Organization. WHO COVID-19 dashboard. [cited May 22, 2024]. Available from: https://data.who.int/dashboards/covid19/cases

[2] World Health Organization. WHO COVID-19 Dashboard. [cited May 22, 2024]. Available from: https://data.who.int/dashboards/covid19/deaths

[3] Pavelka M, Van-Zandvoort K, Abbott S, Sherratt K, Majdan M, CMMID COVID-19 Working Group, et al. The impact of population-wide rapid antigen testing on SARS-CoV-2 prevalence in Slovakia. Science. 2021;372(6542):635–41. doi: 10.1126/science.abf9648 33758017 PMC8139426

[4] Cavazza M, Sartirana M, Wang Y, Falk M. Assessment of a SARS-CoV-2 population-wide rapid antigen testing in Italy: a modeling and economic analysis study. Eur J Public Health. 2023;33(5):937–43. doi: 10.1093/eurpub/ckad125 37500599 PMC10567128

[5] Philippe C, Bar-Yam Y, Bilodeau S, Gershenson C, Raina SK, Chiou S-T, et al. Mass testing to end the COVID-19 public health threat. The Lancet Regional Health – Europe. 2023;25:100574. doi: 10.1016/j.lanepe.2022.100574 36628300 PMC9816799

[6] Zhang X, Barr B, Green M, Hughes D, Ashton M, Charalampopoulos D, et al. Impact of community asymptomatic rapid antigen testing on covid-19 related hospital admissions: synthetic control study. BMJ. 2022;379:e071374. doi: 10.1136/bmj-2022-071374 36418047 PMC9682337

[7] Muttamba W, O’Hare BA-M, Saxena V, Bbuye M, Tyagi P, Ramsay A, et al. A systematic review of strategies adopted to scale up COVID-19 testing in low-, middle- and high-income countries. BMJ Open. 2022;12(11):e060838. doi: 10.1136/bmjopen-2022-060838 36396316 PMC9676418

[8] World Health Organization. COVID-19 Epidemiological Update. May 2024. Available from: https://www.who.int/docs/default-source/coronaviruse/situation-reports/covid_19_epi_update_167.pdf?sfvrsn=58f54395_2&download=true

[9] Han E, Tan MMJ, Turk E, Sridhar D, Leung GM, Shibuya K, et al. Lessons learnt from easing COVID-19 restrictions: an analysis of countries and regions in Asia Pacific and Europe. Lancet. 2020;396(10261):1525–34. doi: 10.1016/S0140-6736(20)32007-9 32979936 PMC7515628

[10] Onyeaka H, Anumudu CK, Al-Sharify ZT, Egele-Godswill E, Mbaegbu P. COVID-19 pandemic: a review of the global lockdown and its far-reaching effects. Science Progress. 2021;104(2):003685042110198. doi: 10.1177/00368504211019854 34061685 PMC10454957

[11] Miles DK, Stedman M, Heald AH. “Stay at Home, Protect the National Health Service, Save Lives”: A cost benefit analysis of the lockdown in the United Kingdom. Int J Clin Pract. 2021;75(3):e13674. doi: 10.1111/ijcp.13674 32790942 PMC7435525

[12] Guzmán Ruiz Y, Vecino-Ortiz AI, Guzman-Tordecilla N, Peñaloza-Quintero RE, Fernández-Niño JA, Rojas-Botero M, et al. Cost-effectiveness of the COVID-19 test, trace and isolate program in Colombia. Lancet Reg Health Am. 2022;6:100109. doi: 10.1016/j.lana.2021.100109 34755146 PMC8560002

[13] Shaw AG, Troman C, Akello JO, O’Reilly KM, Gauld J, Grow S, et al. Defining a research agenda for environmental wastewater surveillance of pathogens. Nat Med. 2023 Sep;29(9):2155–57. doi: 10.1038/s41591-023-02457-7 37537374

[14] Barnes KG, Levy JI, Gauld J, Rigby J, Kanjerwa O, Uzzell CB, et al. Utilizing river and wastewater as a SARS-CoV-2 surveillance tool in settings with limited formal sewage systems. Nat Commun. 2023;14(1):7883. doi: 10.1038/s41467-023-43047-y 38036496 PMC10689440

[15] Hyllestad S, Myrmel M, Lomba JAB, Jordhøy F, Schipper SK, Amato E. Effectiveness of environmental surveillance of SARS-CoV-2 as an early warning system during the first year of the COVID-19 pandemic: a systematic review. Journal of Water and Health. 2022;20(8):1223–42. doi: 10.2166/wh.2022.115 36044191

[16] Lim S, Sohn M. How to cope with emerging viral diseases: lessons from South Korea’s strategy for COVID-19, and collateral damage to cardiometabolic health. Lancet Reg Health West Pac. 2023;30:100581. doi: 10.1016/j.lanwpc.2022.100581 36093123 PMC9442269

[17] Shim E, Tariq A, Chowell G. Spatial variability in reproduction number and doubling time across two waves of the COVID-19 pandemic in South Korea, February to July, 2020. Int J Infect Dis. 2021;102:1–9. doi: 10.1016/j.ijid.2020.10.007 33038555 PMC7543697

[18] Cumming J. Going hard and early: Aotearoa New Zealand’s response to COVID-19. HEPL. 2022;17(1):107–119. doi: 10.1017/S174413312100013X 33663626 PMC8007940

[19] Shrestha S, Yoshinaga E, Chapagain SK, Mohan G, Gasparatos A, Fukushi K. Wastewater-based epidemiology for cost-effective mass surveillance of COVID-19 in low- and middle-income countries: challenges and opportunities. Water. 2021;13(20):2897. doi: 10.3390/w13202897

[20] Hagedorn BL, Gauld J, Feasey N, Hu H. Cost-effectiveness of using environmental surveillance to target the roll-out typhoid conjugate vaccine. Vaccine. 2020;38(7): 1661–70. doi: 10.1016/j.vaccine.2019.12.061 31917040

[21] Ahmed W, Angel N, Edson J, Bibby K, Bivins A, O’Brien JW, et al. First confirmed detection of SARS-CoV-2 in untreated wastewater in Australia: a proof of concept for the wastewater surveillance of COVID-19 in the community. Science of The Total Environment. 2020;728:138764. doi: 10.1016/j.scitotenv.2020.138764 32387778 PMC7165106

[22] Dzinamarira T, Murewanhema G, Iradukunda PG, Madziva R, Herrera H, Cuadros DF, et al. Utilization of SARS-CoV-2 wastewater surveillance in Africa—a rapid review. Int J Environ Res Public Health. 2022;19(2):969. doi: 10.3390/ijerph19020969 35055789 PMC8775514

[23] Fontenele RS, Yang Y, Driver EM, Magge A, Kraberger S, Custer JM, et al. Wastewater surveillance uncovers regional diversity and dynamics of SARS-CoV-2 variants across nine states in the USA. Science of The Total Environment. 2023;877:162862. doi: 10.1016/j.scitotenv.2023.162862 36933724 PMC10017378

[24] Haramoto E, Malla B, Thakali O, Kitajima M. First environmental surveillance for the presence of SARS-CoV-2 RNA in wastewater and river water in Japan. Sci Total Environ. 2020;737:140405. doi: 10.1016/j.scitotenv.2020.140405 32783878 PMC7305903

[25] Randazzo W, Truchado P, Cuevas-Ferrando E, Simón P, Allende A, Sánchez G. SARS-CoV-2 RNA in wastewater anticipated COVID-19 occurrence in a low prevalence area. Water Research. 2020;181:115942. doi: 10.1016/j.watres.2020.115942 32425251 PMC7229723

[26] Sherchan SP, Shahin S, Ward LM, Tandukar S, Aw TG, Schmitz B, et al. First detection of SARS-CoV-2 RNA in wastewater in North America: a study in Louisiana, USA. Sci Total Environ. 2020;743:140621. doi: 10.1016/j.scitotenv.2020.140621 32758821 PMC7833249

[27] Lee BE, Sikora C, Faulder D, Risling E, Little LA, Qiu Y, et al. Early warning and rapid public health response to prevent COVID-19 outbreaks in long-term care facilities (LTCF) by monitoring SARS-CoV-2 RNA in LTCF site-specific sewage samples and assessment of antibodies response in this population: prospective study protocol. BMJ Open. 2021;11(8):e052282. doi: 10.1136/bmjopen-2021-052282 34417219 PMC8382669

[28] Ngwira LG, Sharma B, Shrestha KB, Dahal S, Tuladhar R, Manthalu M, et al. Cost of wastewater-based environmental surveillance for SARS-CoV-2: Evidence from pilot sites in Blantyre, Malawi and Kathmandu, Nepal. PLOS Glob Public Health. 2022;2(12):e0001377. doi: 10.1371/journal.pgph.0001377 36962924 PMC10021894

[29] Ali S, Gudina EK, Gize A, Aliy A, Adankie BT, Tsegaye W, et al. Community wastewater-based surveillance can be a cost-effective approach to track COVID-19 outbreak in low-resource settings: feasibility assessment for Ethiopia context. Int J Environ Res Public Health. 2022;19(14):8515. doi: 10.3390/ijerph19148515 35886369 PMC9319732

[30] Kerr CC, Stuart RM, Mistry D, Abeysuriya RG, Rosenfeld K, Hart GR, et al. Covasim: an agent-based model of COVID-19 dynamics and interventions. PLoS Comput Biol. 2021;17(7):e1009149. doi: 10.1371/journal.pcbi.1009149 34310589 PMC8341708

[31] Wölfel R, Corman VM, Guggemos W, Seilmaier M, Zange S, Müller MA, et al. Virological assessment of hospitalized patients with COVID-2019. Nature. 2020 May;581(7809):465–9. doi: 10.1038/s41586-020-2196-x Epub 2020 Apr 1. 32235945

[32] Verity R, Okell LC, Dorigatti I, Winskill P, Whittaker C, Imai N, et al. Estimates of the severity of coronavirus disease 2019: a model-based analysis. Lancet Infect Dis. 2020;20(6):669–77. doi: 10.1016/S1473-3099(20)30243-7 32240634 PMC7158570

[33] World Bank. GDP per capita (current US$). Available from: https://data.worldbank.org/indicator/NY.GDP.PCAP.CD

[34] Mao Z, Li X, Jit M, Beutels P. COVID-19-related health utility values and changes in COVID-19 patients and the general population: a scoping review. Qual Life Res. 2024;33(6):1443–54. doi: 10.1007/s11136-023-03584-x 38206454

[35] Kohli M, Maschio M, Becker D, Weinstein MC. The potential public health and economic value of a hypothetical COVID-19 vaccine in the United States: use of cost-effectiveness modeling to inform vaccination prioritization. Vaccine. 2021;39(7):1157–64. doi: 10.1016/j.vaccine.2020.12.078 33483216 PMC7832653

[36] Fernandes RRA, Santos MDS, Magliano CADS, Tura BR, Macedo LSDN, Padila MP, et al. Cost Utility of Vaccination Against COVID-19 in Brazil. Value in Health Regional Issues. 2022;31:18–24. doi: 10.1016/j.vhri.2022.01.009 35325693 PMC8935121

[37] Wyper GMA, Assunção RMA, Colzani E, Grant I, Haagsma JA, Lagerweij G, et al. Burden of disease methods: a guide to calculate COVID-19 disability-adjusted life years. Int J Public Health. 2021;66:619011. doi: 10.3389/ijph.2021.619011 34744580 PMC8565264

[38] Cutler DM. The Economic Cost of Long COVID: An Update. [cited 30 January 2024]. Available from: https://scholar.harvard.edu/sites/scholar.harvard.edu/files/cutler/files/long_covid_update_7-22.pdf

[39] Ochalek J, Revill P, Manthalu G, McGuire F, Nkhoma D, Rollinger A, et al. Supporting the development of a health benefits package in Malawi. BMJ Glob Health. 2018;3(2):e000607. doi: 10.1136/bmjgh-2017-000607 29662689 PMC5898345

[40] Revill P, Ochalek J, Lomas J, Nakamura R, Woods B, Rollinger A, Suhrcke M, Sculpher M, Claxton K. Chapter 3: Cost-Effectiveness Thresholds: Guiding Health Care Spending for Population Health Improvement. World Scientific Series in Global Health Economics and Public Policy. 2020, p. 75–97. Available from: Cost-Effectiveness Thresholds: Guiding Health Care Spending for Population Health Improvement | Global Health Economics

[41] Mathieu E, Ritchie H, Rodés-Guirao L, Appel C, Giattino C, Hasell J, et al. Coronavirus pandemic (COVID-19). Ourworldindata.org. Available from: https://Ourworldindata.org/Coronavirus.

[42] Davies NG, Klepac P, Liu Y, Prem K, Jit M, CMMID COVID-19 Working Group, et al. Age-dependent effects in the transmission and control of COVID-19 epidemics. Nat Med. 2020 Aug;26(8):1205–11. doi: 10.1038/s41591-020-0962-9 Epub 2020 Jun 16 32546824

[43] Yoo B-K, Iwamoto R, Chung U, Sasaki T, Kitajima M. Economic evaluation of wastewater surveillance combined with clinical COVID-19 screening tests, Japan. Emerg Infect Dis. 2023;29(8):1608–17. doi: 10.3201/eid2908.221775 37486197 PMC10370838

[44] Vandepitte S, Alleman T, Nopens I, Baetens J, Coenen S, De Smedt D. Cost-effectiveness of COVID-19 policy measures: a systematic review. Value in Health. 2021;24(11):1551–69. doi: 10.1016/j.jval.2021.05.013 34711355 PMC8481648

