# Supplementary material for: Cost-effectiveness of wastewater-based environmental surveillance for SARS-CoV-2 in Blantyre, Malawi and Kathmandu, Nepal: A model-based study
Source: PLOS Glob Public Health. 2025 Apr 24;5(4):e0004439. doi: 10.1371/journal.pgph.0004439 (PMC12021199; doi:10.1371/journal.pgph.0004439)
Supplement: S1 Fig — (DOCX) [file pgph.0004439.s001.docx]

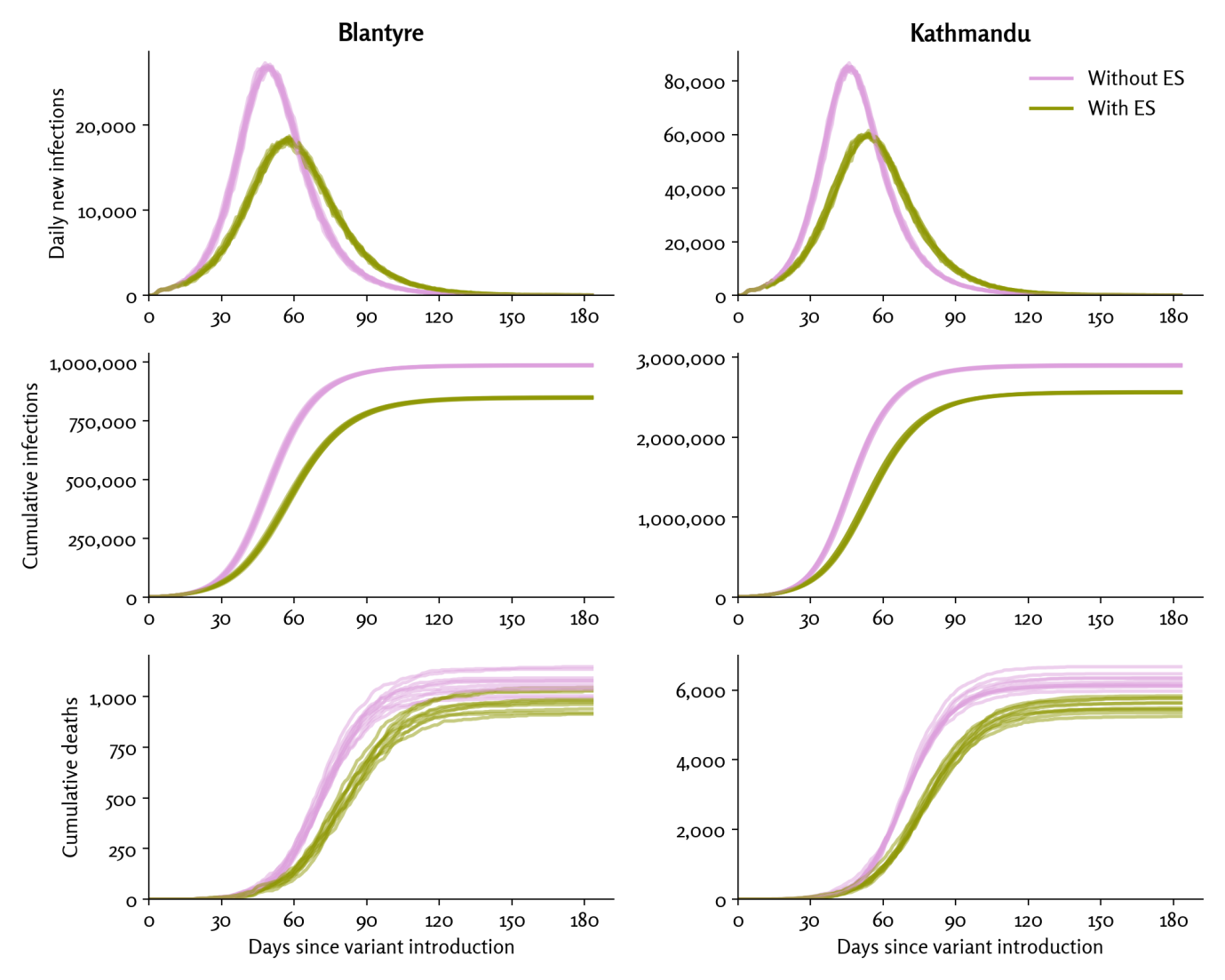


**S1 Fig:** Examples of simulation time series without clinical surveillance with ES (green) and without ES (purple). Compared with the default scenario (which includes clinical surveillance), this scenario shows a much larger difference in the cumulative number of infections and deaths with and without ES.

Abbreviation: ES, environmental surveillance.
